# Variation in the Mu-Opioid Receptor (OPRM1) and Offspring Sex Are Associated With Maternal Behavior in Rhesus Macaques (*Macaca mulatta*)

**DOI:** 10.3389/fnbeh.2022.721958

**Published:** 2022-03-14

**Authors:** Elizabeth K. Wood, Zachary Baron, Melanie L. Schwandt, Stephen G. Lindell, Christina S. Barr, Stephen J. Suomi, J. Dee Higley

**Affiliations:** ^1^Department of Psychology, Brigham Young University, Provo, UT, United States; ^2^Department of Neuroscience, Brigham Young University, Provo, UT, United States; ^3^National Institute on Alcohol Abuse and Alcoholism (NIAAA), Bethesda, MD, United States; ^4^Eunice Kennedy Shriver National Institute of Child Health and Human Development (NICHD), Bethesda, MD, United States

**Keywords:** attachment, infant sex, mother-infant behavior, mu-opioid receptor genotype, rhesus monkeys

## Abstract

A μ-opioid receptor (OPRM1) single-nucleotide-polymorphism, found in both humans and rhesus macaques mediates the mother-infant attachment bond. Because mothers treat their sons and daughters differently, it is somewhat surprising that the role of infant sex has not been assessed in the context of a maternal-OPRM1-genotype-by-infant-sex interaction. The present study investigates the effect of maternal-OPRM1-genotype and infant sex on mother-infant behaviors. Over the first 6 months of offspring life, mother-infant behavioral data assessing attachment quality was collected twice weekly from a large number of rhesus monkey mother-infant pairs (*N* = 161 dyads; *n* = 64 female infants, *n* = 97 male infants). Mothers were genotyped for OPRM1 variation. Factor analysis of the observed behaviors showed two factors: Attachment (maternal-infant cradling, rejections, and infant approaches and leaves), and Maternal Restraints (mother restrains infant, preventing exploration). Further analyses showed a two-way, maternal-genotype-by-infant-sex interaction for both factors. For Attachment, mothers with the CC genotype cradled and restrained (Maternal Restraints) their female infants more and rejected them less, when compared to female infants of CG mothers. Perhaps as a consequence, female infants of CC genotype mothers approached and left their mothers less often, when compared to female infants of CG mothers, likely an indication that female infants from mothers with CG genotype play a greater role in maintaining the mother-infant bond than do female infants from CC genotype mothers. This finding may also indicate a more secure attachment in infants from CC genotype mothers. Unlike female infants, on average, the mother-infant relationship of dyads with a male infant was largely undifferentiated by maternal genotype. These findings suggest that, in contrast to female infants from CG mothers, CC mothers and their female infants appear to have a closer mother-infant relationship which may portend close life-long bonds, as mothers and female offspring remain together throughout life. Male offspring appear to have a more aloof mother-infant bond regardless of OPRM1-genotype. The results of this study indicate that maternal-OPRM1 variation mediates mother-infant attachment behaviors for female infants and has less effect for male infants. This suggests that offspring sex should be included in studies investigating the effect of maternal-OPRM1 genotype on the mother-infant attachment relationship.

## Introduction

In primates, the mother-infant bond is not only necessary for survival, but critical to the normative development of the complex primate brain. Because most brain growth and pruning occur postnatally in primates, appropriate maternal care (i.e., providing the right input at the right time) is important to healthy infant neurological development (Greenough and Black, 1992). Appropriate maternal treatment is largely based on maternal sensitivity to infant arousal, and providing a secure base when needed, ultimately promoting infant self-regulation of arousal (Harlow et al., 1965; Bowlby, 1969). Variation in maternal treatment is largely dependent on an infant’s needs, which vary interindividually, as well as differences in the treatment of male and female infants (Mitchell and Brandt, 1970).

In Maccoby and Jacklin’s (1978) classic monograph of sex differences, they critically review findings showing that male and female infants behave differently beginning early in life. For example, they conclude that infant males are more active, aggressive, and independent than females. Consistent with Maccoby and Jacklin’s (1978) conclusions, in a meta-analysis of 16 studies investigating sex differences in overall infant activity, Campbell and Eaton (1999) found a sex difference in activity, with boys more active than girls. Female infants coo, vocalize, and are more attentive to social cues than are males, although Campbell and Eaton (1999) conclude that this difference in social attention and interactions is small in effect size. Other studies show evidence of increased sociality in females beginning early in life. For example, Goldberg and Lewis (1969) found that females spend more time looking at and touching their mothers, show a shorter latency to return to their mothers, and exhibit increased vocalizations with their mothers. Mothers of infant daughters talk to and look more often at their female infants (Lewis, 1972, p. 106), which may be a function of the infants’ behaviors, with female infants showing higher rates of vocalizations and more time looking at their mothers. Mothers are more likely to seek and maintain close proximity with their infant daughters, when compared to infant sons (Lindahl and Heimann, 1997), and when they are not in close proximity, mothers are more likely to vigilantly watch their daughters than their sons (Lindahl and Heimann, 1997). Studies also indicate that parents allow their sons a higher degree of autonomy to explore, when compared to their daughters (Endendijk et al., 2016). One cannot rule out, however, that sex-linked infant behaviors may elicit these differences in maternal behaviors. For example, when the two sexes were compared in one study (Connellan et al., 2000), male neonates exhibited a stronger interest in a physical-mechanical mobile, whereas female infants showed greater interest in a face (but see Maylott et al., 2021). In non-human primates, female infants reared in a neonatal nursery, where they have no experience with adult monkeys, look longer at computer generated faces and engage in more social affiliative behaviors with their human caregivers than males (Simpson et al., 2016). Sugita (2008) hand reared infant macaques in a neonatal nursery while wearing faceless masks. Despite having no experience with faces, later when the infants were tested, researchers found that compared to males, females preferred to look at faces, whether human or monkey, rather than objects, and one could argue increased interest and looking at the face is likely to elicit caregiver social interactions. Male infants’ greater overall activity, increased rates of play (particularly rough and tumble play), and higher rates of aggression could also affect maternal behaviors, leading to differential treatment of sons and daughters.

Given the relatively large cultural effects on human development, teasing out the origin and effects of sex differences on maternal behavior is difficult. Lonsdorf (2017) and Wood et al. (2020), suggest that non-human primates are useful models to assess the origin and effects of male-female differences on maternal treatment of her offspring. For example, geckering is a non-human primate behavior that very young infants use to elicit maternal solicitude, much like crying in young human infants. Patel and Owren (2007) found that in rhesus macaques, females geckered longer and more often to their mothers, but mothers responded more often to their male offsprings’ geckers. Bentley-Condit (2003) found that in early infancy, mothers initiated more frequent breaks in contact with their sons. Other studies in non-human primates show that mothers were more often in physical contact or close proximity and groomed their daughters more often than their sons (Jensen et al., 1968; Mitchell and Brandt, 1970; Caine and Mitchell, 1979; Nakamichi et al., 1990). Compared to females, male infants are more active, and female infants are more likely to be restrained by their mothers, thus maintaining close proximity (Maestripieri, 1999; Fairbanks, 2003). Rhesus monkey mothers are more likely to promote male independence by rejecting their male infants at an earlier age, when compared to their female infants (Jensen et al., 1968). Taken together, such studies show the utility of non-human primates for the study of sex-mediated maternal behavior differences and the potential of non-human primates to model human mother-infant variation, particularly as it relates to sex differences.

In Lonsdorf’s (2017) comprehensive review of sex differences in infant non-human primates (2017), she concludes, “it is difficult to separate differential maternal behavior from differential infant behavior, which presents a challenging “chicken or egg” problem for such studies.” Put another way, the observed sex differences in maternal treatment may be due to differences in the behaviors of male and female infants, as mothers, for example, were bitten aggressively more often by sons than by daughters (Mitchell, 1968), which may affect maternal behaviors, leading to greater male independence. The tendency for males to exhibit more play and overall activity may in part modulate maternal behaviors that promote independence. Rhesus monkey mothers demonstrate consistent individual differences in maternal style, principally along the dimensions of protectiveness (cradling, approaching, grooming infant) and maternal rejections (Fairbanks’, 1996). In Fairbanks’ (1996) widely cited manuscript on maternal styles, she leaves open the possibility that maternal style may be mediated by the sex of the infant. Consistent with Fairbanks’ (1996) postulates concerning maternal domains of restrictiveness and rejections, greater independence in males may provide more opportunities for mothers to retrieve them when they explore (Lonsdorf, 2017). Conversely, Bentley-Condit (2003) found that female infants are more likely to maintain close social proximity to their mothers when compared to males. Taken together, one possibility is that the behavior of the mother is modulated, at least in part, by the sex-linked behavior of the infant, suggesting that infant sex may create a different environment for the mother.

It is also clear that interindividual differences in neurobiological systems affects the attachment bond. One important CNS system that plays a role in modulating secure base behaviors is the opioid system. Kalin’s widely cited studies assessing infant rhesus monkeys’ response to mother-infant separation show that when compared to placebo, infants administered a non-sedating dose of a μ-opioid receptor agonist, such as morphine, exhibit decreased proximity seeking and distress vocalizations following mother-infant separation; whereas agents that block the μ-opioid receptor, such as naltrexone, reverse the effect, increasing proximity seeking and clinging to mother, as well as distress vocalizations (Kalin et al., 1988, 1995). Important to the hypotheses of this study, rhesus monkey mothers that were administered low dose, non-sedating morphine or heroin also show decreased close intimate proximity with their infants (Misiti et al., 1991; Kalin et al., 1995); whereas administration of the μ-opioid antagonist naltrexone increases social proximity with their infants (Kalin et al., 1995).

In both human and non-human primates, mothers vary in contact seeking with their infants, and studies suggest that inter-individual differences in the response of the opioid system is related to differences in maternal care (Barr et al., 2008; Partington et al., 2018). This variation in maternal behavior is in part due to the degree of response of the opioid system, which is, in part, regulated by differences in the μ-opioid receptor (OPRM1) genotype (Bond et al., 1998; Lee et al., 2011). Rhesus monkeys and humans possess functionally similar non-synonymous OPRM1 single-nucleotide polymorphisms (SNPs) (humans: A118G,Bond et al., 1998; rhesus monkeys: C77G, Miller et al., 2004), resulting in two common genotypes, an ancestral *CC* and a more recent *CG* genotype, and a rare *GG* genotype. Studies indicate that the human *CG* genotype is associated with increased μ-opioid-receptor affinity for β-endorphin *in vitro* (Bond et al., 1998, however, see Beyer et al., 2004; Kroslak et al., 2007), with *in vivo* studies in rhesus monkeys corroborating this, suggesting that the *G* allele SNP confers a gain-of-function, leading to increased reinforcement from endorphins and as a consequence of social affiliation (Miller et al., 2004; Barr et al., 2008).

Behavioral studies in humans and non-human primates have supported the idea that variation in the OPRM1 genotype affects the mother-infant bond and maternal style. Higham et al. (2011) showed that when compared to rhesus monkey mothers homozygous for the *C* allele, mothers with the *G* allele were more likely to keep their infants in close proximity and to restrain them more frequently. Barr et al. (2008) showed that during periods of mother-infant social separation, infants carrying the *G* allele exhibited greater rates of distress vocalizations and spent more time in close intimate proximity with their mothers during reunions, when compared to infants homozygous for the *C* allele (Barr et al., 2008). These findings suggest that the *G* allele is associated with increased intimate mother-infant social proximity and secure base seeking in infants. Since the landmark discoveries that genotypic effects are dependent on environmental input (gene × environment interactions; Bennett et al., 2002; Caspi and Moffitt, 2006), researchers have increased their focus on environmentally dependent genotypic influences (Zhu et al., 2009). As noted earlier, in the context of mother-infant interactions, one important element of the mother’s environment is the behavioral differences observed between male and female infants (Lonsdorf, 2017). Increased male infant aggression, play, and overall activity, and increased social proximity seeking in female infants, may create different environments for mothers rearing their offspring. This suggests the possibility that the behavior of the mother is modulated, at least in part, by the sex-linked behavior of the infant. Male-female differences are observed early in life, which lead to sex-dependent differences in the mother’s environment and ultimately her behavior. Thus, it is surprising that studies have not assessed the effect of the μ-opioid-receptor variation on maternal behavior in the context of infant sex and ultimately, the different environments that male and female infants create.

Rhesus macaques are ideally suited to model maternal genetic influences on the differential treatment of their male and female infants. Among non-human primates, the mother-infant relationship has been best characterized in rhesus macaques (Maestripieri, 2005; Maestripieri et al., 2009), with Harlow’s early studies (Harlow and Zimmermann, 1959; Harlow et al., 1965) laying the groundwork for Bowlby’s (1969) development of attachment theory (Suomi et al., 2008). As fellow primates, they share a large degree of genetic similarity with humans, making them well-suited to model genotypic influences. Importantly, unlike humans, they can be reared in standardized, homogenous environments, increasing the capacity to maintain control over confounding variables and consequently increase the capacity to detect candidate gene influences.

This study focuses on the effects of maternal-OPRM1 genotype and infant sex on mother-infant behavior over the first 5 months of infant life, assessing mother-infant behaviors that characterize attachment and independence. Given the previously observed maternal OPRM1 genotype-mediated and infant sex-dependent behavioral differences described above, the study hypothesizes an infant-sex-by-maternal genotype interaction. Specifically, the study has two primary aims. First, the study investigates the relationship between maternal OPRM1 genotype and offspring sex on mother-infant attachment. Given that female infant macaques remain with their natal family group throughout life and show evidence of stronger social bonding with their mothers when compared to males, it is hypothesized that, when compared to mothers with the more recent *CG* genotype, mothers with the ancestral *CC* genotype will exhibit more secure attachment-like behaviors with their daughters. Specifically, mothers with the *CC* genotype will exhibit more maternal mutual ventral cradling, more maternal restraints, and less frequent rejections of their female infants. Because daughters of *CC* genotype mothers spend more time in mutual ventral cradling and are restrained by their mothers more frequently, their female infants will show fewer departures from and approaches to their mothers, when compared to female infants whose mothers possess the more recent and less frequent *CG* genotype. Second, the study investigates the relationship between maternal OPRM1 genotype and offspring sex on maternal restrictiveness and infant independence. Because studies show that males are more focused on peer-interactions, show greater overall activity, and exhibit more frequent aggression than females, it is predicted that male infants will be less affected by maternal genotype than female infants, that they will show similar patterns of independence regardless of maternal genotype, and that their mothers will show similar lower rates of restraints regardless of genotype.

## Materials and Methods

### Subjects

Subjects were *N* = 161 rhesus monkey mother-infant dyads (*n* = 64 female offspring, *n* = 97 male offspring) housed in indoor-outdoor enclosures at the National Institutes of Health Animal Center in Poolesville, Maryland. The colony is ideal for genetic studies, as the total pedigree could be traced back to the founders that originated from distal locations, making them unlikely to be related. Outbreeding was maintained to prevent relatedness, and within colony genetic diversity was maintained using planned matings. Acquisition of new sires and dams was also based on non-relatedness to subjects in the colony, showed that the average identity-by-descent is 1.68%, approximately equivalent to the relatedness of third cousins, which previous studies have used as an acceptable criterion for an outbred pedigree (Robin et al., 1997; Newman et al., 2005). Within colony genetic diversity was maintained using planned matings.

Mother-infant dyads were housed in mixed-sex social conditions approximating the natural rhesus monkey social setting (with 1–2 adult males and 7–10 adult females with their infant offspring), albeit smaller in number than the average wild rhesus monkey troop (Lindburg, 1971). Subjects were housed in indoor-outdoor enclosures (indoor: 2.44 × 3.05 × 2.21 m; outdoor 2.44 × 3.0 × 2.44 m). The sample included infants from seven birth-year cohorts from 1991 through 2005. Water was provided *ad libitum*, and monkeys were fed a diet of Purina^®^ High Protein Monkey Chow (#5038) twice a day (early morning well before data collection, and late afternoon following data collection). Chow was supplemented with fresh fruit three times a week and sunflower or other seeds were provided daily. Fruits, seeds, and other environmental enrichment were provided after behavioral coding. Protocols for the use of experimental animals were approved by the Institutional Animal Care and Use Committee of the National Institute on Alcohol Abuse and Alcoholism.

### OPRM1 Genotyping

Blood was obtained and DNA was extracted for OPRM1 genotyping of the mothers. Genotyping procedures are described in detail elsewhere (see Barr et al., 2008). Briefly, DNA was isolated from whole blood collected from the femoral vein after subjects were sedated (ketamine anesthesia, 15 mg/kg, IM). A portion of the OPRM1 exon was amplified from 25 μg of genomic DNA using AmpliTaq Gold and 2.5 mM MgCl_2_ according to manufacturer’s instruction (Invitrogen). Samples were separated by electrophoresis on 10% polyacrylamide gels, and the *C* and *G* alleles were identified by direct visualization after ethidium bromide staining (*n* = 109 mothers were homozygous for the *C* allele, *n* = 48 mothers possessed one copy of the *G* allele, *n* = 4 mothers were homozygous for the *G* allele [Given the low genetic availability of individuals homozygous for the *G* allele, for the purpose of analysis, those heterozygous for the *G* allele were combined with those homozygous for the *G* allele resulting in a *n* = 52 carriers of the *G* allele)]. Genotype frequency did not deviate from Hardy–Weinberg equilibrium [χ^2^(2, *N* = 159) = 5.53, *p* = 0.84]. See Table 1 for a distribution of maternal genotype and offspring sex.

**TABLE 1:** Frequency of maternal OPRM1 genotypes by offspring sex.

	CC	CG
Female	41	23
Male	68	29

### Behavioral Data Collection

For the first 5 months of infant life, 5-min focal observations were conducted by trained observers twice a week, providing 48 behavioral coding sessions using an objectively defined, mutually exclusive, exhaustive scoring system. Behaviors were recorded in the outdoor portion of the indoor-outdoor runs between 1,300 and 1,500, with subject order randomly assigned. Planned medical treatments and feedings did not occur during that time, and subjects in cages where medical treatments had occurred in the morning were scored the next day. Clear plexiglass and a 1.9 cm wire mesh barrier separated the outdoor adjacent runs. Behavioral coders paused scoring the monkeys when fights or loud distractions occurred. Because the behavior coders scored subjects in adjacent runs daily, subjects were well-habituated to the presence of the behavior coders. All coders were trained by a senior investigator with extensive experience in behaviorally coding rhesus monkeys (SGL) and all observers achieved an inter-rater reliability of 85% or higher, with additional assessments of reliability performed twice yearly to maintain consistent behavioral coding across time. Focal behavioral observations included behaviors that are indicative of the mother-infant attachment relationship in rhesus monkey, using an ethogram developed and used extensively by the senior author’s laboratory (Barr et al., 2008). See Table 2 for a list of behaviors and definitions. The study was a multi-year project, and one behavior (maternal restraints) was added in the fourth year of the study, limiting the number of subjects for the behavior maternal restraint to *n* = 81.

**TABLE 2:** Mother-infant ethogram with behavioral definitions.

Behavior	Definition
Mother approaches Infant approaches	Mother initiates an approach toward the infant until she is within an arm’s length in proximity Infant initiates an approach toward the mother until the infant is within an arm’s length in proximity
Mother leaves	Mother initiates leaving the infant and is no longer within proximity (less than an arm’s length in proximity)
Infant leaves Mother groom	Infant initiates leaving the mother and is no longer within proximity (less than an arm’s length in proximity) Mother scratches, licks, or spreads her offspring’s fur with her fingers or mouth
Mutual-ventral contact	The ventrum of the mother and the offspring are touching. Infant may or may not have nipple in mouth. Infant cannot be sleeping (eyes closed for more than 30 s)
Reject infant	Mother refuses her infant’s attempts to approach or make contact by turning, blocking access to the nipple, pushing, or pulling the infant away from or off her body
Restrain infant	Mother grabs, holds, or tugs at infant attempting to leave her proximity
Social contact	Mother is in physical contact or in arm’s length of her infant, but the ventrum of the mother and the offspring are not touching

*The mother is the focal subject, but incidents of approach and leave by the infant were also recorded. All behaviors were recorded as average frequency (per 5 min), with the exception of mutual ventral contact and social contact which were recorded as average time in seconds (per 5 min).*

### Data Analysis

Preliminary analyses showed no effect of cohort year and thus it was not included in the analyses. Further preliminary analyses of infant age (month of life) did not interact with maternal genotype or infant sex across the 6 months of the study. Therefore, repeated measures were not employed. Funder (1983) showed that aggregating data across repeated sampling reduces variability, increases power and behavioral trait stability. Hence the data for each behavior were averaged across the first 5 months and the mean value for each behavior was used as the dependent variable. Further analyses showed that some of the behaviors were positively correlated with one another (see Supplementary Appendix 1). Therefore, factor analysis was therefore used to assess for underlying source variables (Supplementary Appendix 2) using pairwise deletion. The factorability of the behaviors was assessed, with results indicating that the data was well-suited for factor analysis (Kaiser-Meyer-Olkin Measure of Sampling Adequacy: KMO = 0.69; Bartlett’s Test of Sphericity: [χ^2^(10) = 154.02; *p* < 0.001]. Principal axis factoring with varimax rotation was used to assess for underlying dimensions. Because the behavior maternal restraints was only collected on 74 subjects, to utilize the full sample, two separate factor analyses were performed. The first included all of the subjects and behaviors, except restraints (*n* = 149), and for the second factor analysis (KMO = 0.71; Bartlett’s Test of Sphericity: [χ^2^(6) = 337.60; *p* < 0.001] restraints were added (*n* = 74).

For the first factor analysis, only one factor emerged, consisting of the behaviors, infant approaches, infant leaves, mutual ventral cradling and rejections, explaining 65.49% of the variance. All of the behaviors except mutual ventral cradling loaded positively. For the second factor analysis that included the behavior restraints, two factors emerged, with Factor 1 consisting of the same behaviors and directionality of factor loading as the first factor analysis, although the factor loading scores were slightly different (see Supplementary Appendix 2). Factor 2 consisted of only one behavior, maternal restraints (Supplementary Appendix 2 for the factor loading matrix), with the second factor analysis explaining 74.65% of the variance. Like factor analysis 1, there was a negative loading for mutual ventral contact, and positive loadings for infant approaches and leaves, and maternal rejections, this factor was conceptualized as *Attachment*. Because mutual ventral cradling is commonly thought to characterize attachment, to facilitate interpretability the factor scores for the *Attachment* factor were reversed scored by multiplying the factor scores by –1. The second factor included only restraints (the factor was therefore conceptualized as *Maternal Restraints*), which exhibited a negative loading. Maternal grooming did not load well on either factor (<0.2). Thus, it was not included in the final solution. The factor scores for both factors were utilized as the dependent variables in the planned analyses.

To test the effect of maternal OPRM1 genotype and infant sex on *Attachment* and *Maternal Restraints*, two-way between groups ANOVAs were performed with maternal OPRM1 genotype (*CC* or *CG*) and infant sex (male or female) as the independent variables. As preliminary analyses using ANCOVA showed that maternal age made a significant contribution as a covariate, maternal age was retained in the models. As neither parity nor rank contributed to the effects on the variables of interest (*p* > 0.05), they were dropped from the ANCOVAs. Levene’s test for normality showed that neither of the two factors violated normality across groups. Bonferroni corrections were used to control for the number of *a posteriori* comparisons. All analyses were conducted in SPSS, version 27.

Because a portion of the dams contributed more than one infant to the study (*n* = 64), the models were rerun as linear mixed models, which accounts for repeated measures. The first linear mixed model assessed the relationship between maternal OPRM1 genotype and infant sex (fixed factors) and *Attachment*, with dam ID as a random factor. The second linear mixed model assessed the relationship between maternal OPRM1 genotype and infant sex (fixed factors) and *Maternal Restraints*, with dam ID as a random factor. Each of the models were adjusted for maternal age and Bonferroni corrections were utilized to control for the number of *a posteriori* comparisons between groups.

To assure that overall relatedness was not the driving influence on the OPRM1 genotypic effects, relatedness was calculated using a variance components method developed to investigate genetic effects on a phenotype (Sequential Oligogenic Linkage Analysis Routines (SOLAR; Texas Biomedical Research Institute, San Antonio, TX, Almasy and Blangero, 1998). Using a pedigree matrix, SOLAR calculates the percentage of phenotypic variance that is attributable to additive genetic differences among subjects, and as in the ANCOVA models, maternal age was statistically controlled. The NIH Animal Center colony pedigree is traced back to the original founders, which allows SOLAR to calculate pairwise relatedness values for all subjects in the dataset. This allows a comparison of the variance accounted for with and without the relatedness component, assessing whether OPRM1 genotypic contribution could be explained by overall relatedness. To facilitate comparison of the effect sizes between the models that did and did not account for heritability, all ANCOVA models were rerun as linear regressions.

## Results

### Maternal OPRM1 Genotype and Mother-Infant Attachment

**FIGURE 1:**
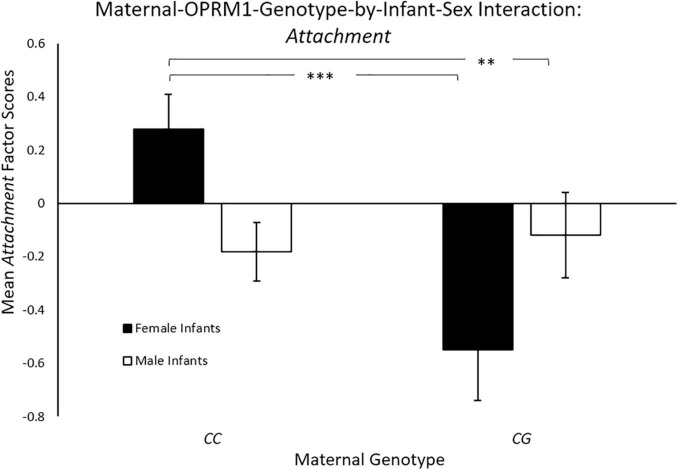
Results showed a significant two-way sex-by-maternal-genotype interaction for Attachment (*p* < 0.001), with CC mothers with daughters exhibiting higher Attachment scores on average, when compared to CG mothers with daughters (p = 0.014) or to CC mothers with sons (*p* = 0.69). White bars represent mothers with male offspring, and black bars represent mothers with female offspring; *p < 0.05, **p < 0.01, and ***p < 0.001.

Controlling for maternal age, there was a significant main effect for maternal genotype [*F*(1, 144) = 6.35, *p* = 0.013; η*_*p*_*^2^ = 0.04],with mothers with the *CC* genotype exhibiting significantly higher mean *Attachment* scores (*M* = 0.11 ± 0.09), when compared to mothers with the *CG* genotype (*M* = –0.27 ± 0.13). There was also a significant two-way maternal-OPRM1-genotype-by-infant-sex interaction for mean *Attachment* factor scores [*F*(1, 144) = 8.70, *p* = 0.004; η*_*p*_*^2^ = 0.06, see Figure 1]. *CC* mothers with female infants exhibited higher *Attachment* scores (*M* = 0.35 ± 0.13 *p* < 0.001), when compared to *CG* mothers of female infants (*M* = –0.49 ± 0.20), or to *CC* mothers with male infants (*M* = –0.12 ± 0.11; *p* = 0.007). The mean *Attachment* factor scores of mothers with a male infant were undifferentiated by maternal genotype (*p* < 0.05).

### Maternal OPRM1 Genotype and Maternal Restraints

**FIGURE 2:**
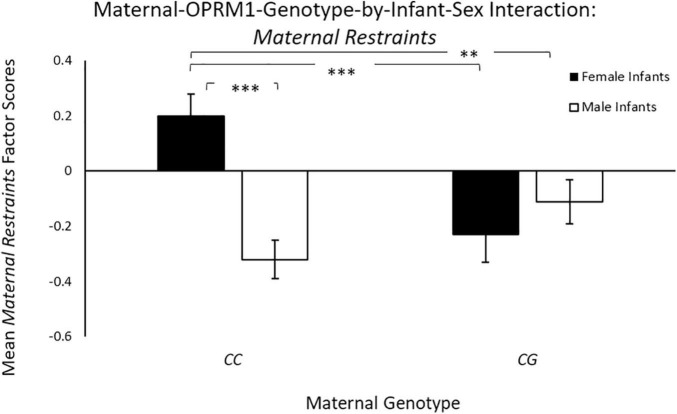
Results showed a significant two-way sex-by-maternal-genotype interaction for Maternal Restraints (p = 0.001), with CC mothers with daughters exhibiting higher Maternal Restraint scores on average, when compared to CG genotype mothers with daughters (p < 0.001) or to CC mothers with sons (*p* < 0.001). White bars represent mothers with male offspring, and black bars represent mothers with female offspring; *p < 0.05, **p < 0.01, and ***p < 0.001.

Controlling for maternal age, there was a significant main effect of infant sex [*F*(1, 65) = 4.07, *p* = 0.04; η*_*p*_*^2^ = 0.06], with mothers with female infants exhibiting higher mean *Maternal Restraints* scores (*M* = 0.14 ± 0.10), when compared to mothers of male infants (*M* = –0.12 ± 0.08). There was also a significant two-way maternal-OPRM1-genotype-by-infant-sex interaction for mean *Maternal Restraints* factor scores [*F*(1, 65) = 6.91, *p* = 0.01; η*_*p*_*^2^ = 0.10; see Figure 2], with *CC* mothers with female infants exhibiting higher factor scores for mean *Maternal Restraints* (*M* = 0.31 ± 0.13), when compared to *CG* mothers with female infants (*M* = –0.29 ± 0.11), or to *CC* mothers with male infants (*M* = –0.29 ± 0.11; *p* < 0.001), or to *CG* mothers with male infants (*M* = –0.05 ± 0.13; *p* = 0.04).

### Linear Mixed Models

Linear mixed models controlling for multiple offspring from the same dam showed essentially the same results as the ANOVA models. Controlling for maternal age and repeated measurement of dams, there was a significant main effect for maternal genotype on *Attachment* [*F*(1, 81.83) = 4.39, *p* = 0.04], with mothers possessing the *CC* genotype exhibiting significantly higher mean *Attachment* scores (*M* = 0.20 ± 0.21), when compared to mothers possessing the *CG* genotype (*M* = –0.028 ± 0.15). There was also a significant maternal-OPRM1-by-infant-sex interaction [*F*(1, 133.50) = 11.63, *p* < 0.001]. *CC* genotype mothers with female infants exhibited significantly higher mean *Attachment* scores (*M* = 0.36 ± 0.14, *p* < 0.001), when compared to *CG* genotype mothers with female infants (*M* = –0.53 ± 0.20), or to *CC* genotype mothers with male infants (*M* = –0.15 ± 0.12; *p* = 0.003). Mothers possessing the *CG* genotype whose infants were female exhibited higher attachment scores, when compared to mothers possessing the *CG* genotype whose infants were males (*M* = –0.02 ± 0.21; *p* = 0.04). The mean *Attachment* factor scores of mothers with male infants were undifferentiated by maternal genotype).

Controlling for maternal age and repeated measurement of dams, there was a significant main effect for infant sex on *Maternal Restraint* [*F*(1, 66) = 4.03, *p* = 0.04], with mothers of female infants exhibiting significantly higher mean *Maternal Restraint* scores (*M* = 0.14 ± 0.10), when compared to mothers possessing the *CG* genotype (*M* = –0.12 ± 0.08). There was also a significant maternal-OPRM1-by-infant-sex interaction [*F*(1, 66) = 6.94, *p* = 0.01]. *CC* mothers with female infants exhibited higher mean *Restraint* scores (*M* = 0.32 ± 0.13, *p* < 0.001), when compared to *CG* mothers of female infants (*M* = –0.05 ± 0.16), or to *CC* mothers with male infants (*M* = –0.28 ± 0.10; *p* < 0.001), or to *CG* mothers with male infants (*M* = 0.03 ± 0.13).

### Relatedness

The genotypic effects were robust when overall relatedness was controlled for: the variance components attributable to the main effect of the OPRM1 genotype and the maternal-genotype-by-infant-sex interaction remained significant whether or not relatedness was included as a covariate (*h^2^r*) (*p* < 0.001). The estimates of maternal OPRM1 genotype, infant sex, and maternal-OPRM1-genotype-by-infant-sex interaction remained relatively similar to the models that did not include a heritability estimation (see Tables 3, 4).

**TABLE 3:** Attachment.

Regression model 1		
Parameter	*Beta*	*p*
Maternal OPRM1 genotype	–0.41	***
Infant sex	–0.27	**
Maternal OPRM1 genotype * infant sex	0.36	**
Maternal age	–0.28	***
**SOLAR Model 1: *h^2^r* constrained to zero**		
*h^2^r*	0.00	*N.S.*
Maternal OPRM1 genotype	0.27	***
Infant sex	–0.14	***
Maternal OPRM1 genotype * infant sex	0.32	***
Maternal age	0.07	***
**SOLAR Model 2: *h^2^r* estimated**		
*h^2^r*	0.88	*N.S.*
Maternal OPRM1 genotype	0.27	***
Infant sex	–0.15	***
Maternal OPRM1 genotype * infant sex	0.36	***
Maternal age	0.07	***

This table displays coefficients for models assessing the impact of maternal OPRM1 genotype and offspring sex on Attachment. Maternal age was controlled in all models. Models have been presented with and without accounting for heritability (h^2^r). **p < 0.01, ***p < 0.001.

**TABLE 4:** Maternal restraint.

Regression model 2		
Parameter	*Beta*	*p*
Maternal OPRM1 genotype	–0.29	*N.S.*
Infant sex	–0.47	***
Maternal OPRM1 genotype * infant sex	0.44	*
Maternal age	0.12	*N.S.*
**SOLAR Model 2: *h^2^r* constrained to zero**		
*h^2^r*	0.99	*N.S.*
Maternal OPRM1 genotype	–0.09	***
Infant sex	0.09	***
Maternal OPRM1 genotype * infant sex	0.07	***
Maternal age	0.04	***
**SOLAR Model 2: *h^2^r* estimated**		
*h^2^r*	0.99	*N.S.*
Maternal OPRM1 genotype	–0.08	*N.S.*
Infant sex	0.10	*
Maternal OPRM1 genotype*infant sex	0.11	*
Maternal age	0.05	*

This table displays coefficients for models assessing the impact of maternal OPRM1 genotype and offspring sex on Maternal Restraint. Maternal age was controlled in all models. Models have been presented with and without accounting for heritability (h^2^r). *p < 0.05, ***p < 0.001.

## Discussion

Overall, the results indicated that mothers that possessed the *CC* genotype showed evidence of a more engaged mother-infant bond with their daughters as indicated by their *Attachment* factor scores (more mother-infant mutual ventral cradling and fewer maternal rejections of their daughters, and their daughters exhibited fewer approaches and leaves), when compared to *CG* mothers and their female infants. As predicted, male infants were undifferentiated by maternal genotype. For the second factor, *Maternal Restraints*, consistent with a more engaged pattern of maternal behavior, mothers possessing the *CC* genotype restrained their daughters more often than *CG* mothers restrained their daughters. They also restrained their daughters more often than sons from both *CC* and *CG* genotype mothers. Sons were statistically undifferentiated by maternal genotype for the *Attachment* factor, and while the comparison between *CC* and *CG* mothers with sons failed to achieve statistical significance, there was a trend for *CC* mothers to restrain their sons more often than mothers with the *CG* genotype, but it is of note that mothers with either genotype restrained their sons less often than the overall average. These findings suggest that maternal OPRM1 genotype mediates maternal treatment of infant daughters, as well as the behavior of daughters. Maternal OPRM1 genotype was not relevant to the mother-infant attachment bond for males. Overall, the findings suggest that there is an infant-sex-by-maternal-OPRM1 genotype interaction for mother-infant relations and that the effect appears to be more salient for daughters than for sons. When the linear mixed model analyses were employed, the results were essentially identical, an indication that the mother and infant effects were not related to some of the mothers having more than one offspring. This also illustrates the robustness of our findings, with the two types of analyses showing an almost identical set of outcomes.

These results indicate that mothers with the ancestral *CC* genotype treat their male and female infants differently. Daughters from mothers with the *CC* genotype were cradled and restrained more, and perhaps as a consequence of their maternal treatment, female infants from *CC* mothers left and approached their mothers less often. Daughters from *CC* mothers were also rejected less when compared to male infants of either maternal genotype. The behavior of infants from mothers with the ancestral *CC* genotype is consistent with earlier findings showing that infant males are more independent; whereas, infant females exhibit more time in close proximity to their mothers, an indication of a more closely bonded relationship between *CC* mothers and daughters than between mothers and sons (Jensen et al., 1968; Mitchell and Brandt, 1970; Caine and Mitchell, 1979; Nakamichi et al., 1990; Bentley-Condit, 2003; Maylott et al., 2021). Differences in the behavior between the male and female infants from *CC* genotype mothers also portents the strength of future social bonds with the mother. Young adult male rhesus monkeys leave their natal social group (and mother) to migrate to new social groups, whereas, daughters remain with their mothers throughout life, showing a life-long close bond with their mothers. Male infants from *CC* and *CG* mothers show patterns of behavior that are quite similar, whereas daughters from mothers with the *CG* allele do not follow the typical pattern of close bonding with their mothers. They are instead, seldom cradled or restrained, rejected more by their mothers and they show a pattern of increased approaches and leaves. This is a pattern that Hinde and Atkinson (1970) would describe as a mother-infant relationship where the infant is principally responsible for maintaining the relationship.

To the extent that the *CC* genotype represents the ancestral norm, the *CC* mother-infant relationship may be based on a reward system that operates to maintain social bonds reflective of species-specific sex differences in long-term social bonding. Previous studies show an overall male-female difference in sociality that is generally consistent with the findings from this study, with females spending more time in affiliative behaviors than males (Jensen et al., 1968; Timme, 1995; Bentley-Condit, 2003; Simpson et al., 2016). Given the selective advantages of strong bonds between related females in a kin-based matriline, this sex difference may be evolutionarily based. In kin-based matrilineal societies such as rhesus macaques, females remain in their natal group with their mothers and other female kin throughout life (Thierry, 2007), and female kin show coalitional defense of one another and their offspring, whereas males migrate shortly after puberty (Howell et al., 2007; Hayakawa and Soltis, 2011), as indicated earlier. Taken together, the results of previous studies and the findings from this study suggest that female mother-infant preferences for social affiliation and close proximity are modulated, at least in part, by genotype.

In human studies, research has primarily focused on the OPRM1 genotype of the child, and findings show that children with the *G* allele are more likely to form fearful attachments with their parents (Troisi et al., 2011). In one study, for example, researchers focused on the interaction between parental overcontrol and the child’s OPRM1 genotype. The study showed that children with the *G* allele exhibited more sympathetic nervous system reactivity to the stress of maternal overcontrol, when compared to children homozygous for the *C* allele (Partington et al., 2018). These results highlight the significance of OPRM1 genotype on the development of a quality attachment bond between children and parents, particularly for daughters. The present study provides further insight into the impact of maternal genotypic variation on the attachment relationship. One potential gap in the human literature stems from the failure to include the sex of the infant as a potential mediator (i.e., an infant-sex-by-genotype assessment) on the attachment relationship and our results suggest that this is a variable that should be included in future studies.

The findings from this study are largely consistent with the studies in humans cited above, but are not consistent with the Higham et al. (2011) study of rhesus monkeys. Unlike (Higham et al., 2011), the data from this study showed no overall genotype main effects for restraints. Higham et al. (2011) studied a free-ranging group of mothers and found that mothers with the *G* allele restrained their infants more often than *CC* genotype mothers. This may represent Higham and colleagues’ relatively small sample size, which precluded analyses by infant sex. Moreover, they studied their infants well after weaning (some at 9-months of age), and post-weaning mother-infant interactions are more focused on maintaining independence, rather than the formation and maintenance of attachment bonds (Suomi, 2005). In the present study, all infants were studied at the same age and before weaning was complete. It is noteworthy, however, that the percentage of mothers with the *G* allele was much higher in Higham et al., population (56%) than in this study (32%). The population studied by Higham et al. (2011) is from a founder population of about 400 subjects that has undergone several genetic bottlenecks. While the heterozygosity is similar to other populations and inbreeding has been minimized by the introduction of new males, 90% of the subjects from the free-ranging population in the Higham et al. (2011) study are descended from just 15 mothers that were alive during the 1950s (Kanthaswamy et al., 2017). Although somewhat speculative, this may have affected the frequency of the G allele in the population, which may have led to differences in maternal behavior in the Higham et al. (2011) population. Alternatively, the differences in maternal behaviors seen in Higham et al. (2011) study may be a result of long-term cultural transmission (Kanthaswamy et al., 2017).

Attachment quality is based on sensitive parenting behavior that is largely dictated by both the short- and long-term needs of the offspring, with parents adjusting their behavior to fit their infant’s needs. Studies suggest that mother-infant social bonds are maintained for both sexes in part by the opioid system (Kalin et al., 1995), as well as other central neurotransmitter systems (e.g., oxytocin, serotonin, etc., Maestripieri et al., 2009; Mileva-Seitz et al., 2011; Bisceglia et al., 2012). For rhesus macaque males, who leave their family group as young adults, there is less necessity for long-term social bonding with their mothers and the females from their matriline. For females, on the other hand, maintenance of social bonds, particularly with female kin, is the long-term biological and psychological imperative, as they are allies during challenges by females from other matrilines. Most developmental studies investigating sex differences in the acquisition of social competence reflect this sex difference (Brown and Dixson, 2000), with males spending less time grooming and in close social proximity with kin (Maestripieri and Hoffman, 2012). Females, on the other hand, are more likely to groom and to exhibit close social proximity with their kin than are males (Maestripieri and Hoffman, 2012). The sizable *CG* genotype frequency in this study and others may reflect a selective purpose in the maintenance of the *G* allele in the population. While somewhat speculative, from an evolutionary perspective, depending on the environmental demands or the size of a matriline, it likely pays off to allow increased or decreased offspring independence. For example, in a large, high-ranking matriline, increased exploration and independence allows offspring to form more social bonds or explore the environment for unknown resources. On the other hand, in ecological niches filled with predators, or in a small, low-ranking matriline, the mother’s increased vigilance provides a layer of protection that could prove essential to the survival of her offspring. Thus, differences in the μ-opioid system may provide a selective evolutionary advantage depending on ecological and social demands.

This study assessed the effect of OPRM1 genotypic variation on mother-infant behavior, comparing the maternal genotype-specific treatment of male and female infants over the first 5 months of infant life, a developmental period that largely occurs before weaning, in which quality parenting has important consequences for later life outcomes. Developmental studies assessing the differential effects of maternal OPRM1 genotype on the treatment of infant sons and daughters are limited, despite established sex differences in male and female infant behavior. The developmental period covered in this study is limited to infancy, and the authors of this research know of no studies that have investigated the long-term outcomes of maternal OPRM1 genotype on infant development, both within and between the sexes. As infants inherit their parents’ alleles, it is probable that some of the infants have similar alleles as their mothers, and one drawback to this study is that the sample size was too small to assess mother-genotype-by-infant genotype interactions, an important research topic for future studies. The data from this study indicate that offspring sex is also a variable that should be considered in understanding OPRM1 genotypically-mediated parental differences in the treatment of male and female offspring.

## Data Availability Statement

As the data are part of a larger dataset owned and archived by the NIH, the raw data supporting the conclusions of this article will be made available by the authors, with permissions from the NIH, upon request.

## Ethics Statement

The animal study was reviewed and approved by the Institutional Animal Care and Use Committee of the National Institute on Alcohol Abuse and Alcoholism.

## Author Contributions

EW, ZB, and JH contributed to the conception and design of the study, conducted data analyses, interpreted the findings, and wrote the first draft of the manuscript. MS, SL, CB, SS, and JH contributed to the acquisition of the data. EW, ZB, MS, SL, CB, SS, and JH contributed to manuscript revision, critically reviewed content, and approved the final version of the manuscript. All authors contributed to the article and approved the submitted version.

## Conflict of Interest

The authors declare that the research was conducted in the absence of any commercial or financial relationships that could be construed as a potential conflict of interest.

## Publisher’s Note

All claims expressed in this article are solely those of the authors and do not necessarily represent those of their affiliated organizations, or those of the publisher, the editors and the reviewers. Any product that may be evaluated in this article, or claim that may be made by its manufacturer, is not guaranteed or endorsed by the publisher.
