# Supplementary material for: Variation in the Mu-Opioid Receptor (OPRM1) and Offspring Sex Are Associated With Maternal Behavior in Rhesus Macaques (Macaca mulatta)
Source: Front Behav Neurosci. 2022 Mar 14;16:721958. doi: 10.3389/fnbeh.2022.721958 (PMC8964435; doi:10.3389/fnbeh.2022.721958)
Supplement: Supplementary file 1 [file Data_Sheet_1.pdf]

Supplementary Tables

Table 1. *Attachment*

Model 1: h^2r constrained to zero		
Parameter	*Beta*	*p*
h^2r	0.00	*N.S.*
maternal OPRM1 genotype	0.25	***
infant sex	-0.11	***
maternal OPRM1 genotype*infant sex	0.30	***
**Model 2: h^2r estimated**		
Parameter	*Beta*	*p*
h^2r	0.43	*N.S.*
maternal OPRM1 genotype	0.25	***
infant sex	-0.12	***
maternal OPRM1 genotype*infant sex	0.31	***

Note. *$p < .05$, **$p < .01$, ***$p < .001$

Table 2. *Restraint*

Model 1: h^2r constrained to zero		
Parameter	*Beta*	*p*
h^2r	0.00	*N.S.*
maternal OPRM1 genotype	-0.11	***
infant sex	0.09	***
maternal OPRM1 genotype*infant sex	0.05	***
**Model 2: h^2r estimated**		
Parameter	*Beta*	*p*
h^2r	0.92	*N.S.*
maternal OPRM1 genotype	-0.11	***
infant sex	0.11	***
maternal OPRM1 genotype*infant sex	0.07	***

Note. *$p < .05$, **$p < .01$, ***$p < .001$
