# Supplementary material for: Variation in the Mu-Opioid Receptor (OPRM1) and Offspring Sex Are Associated With Maternal Behavior in Rhesus Macaques (Macaca mulatta)
Source: Front Behav Neurosci. 2022 Mar 14;16:721958. doi: 10.3389/fnbeh.2022.721958 (PMC8964435; doi:10.3389/fnbeh.2022.721958)
Supplement: Supplementary file 2 [file Data_Sheet_2.PDF]

Appendix 1

Appendix 1. Correlation matrix

variable	1	2	3	4	5	6
approaches by infant	-					
leaves by infant	.83***	-				
rejections by mother	.44***	.34***	-			
restraints by mother	-.09	-.01	-.17	-		
grooming by mother	-.15	-.17*	-.05	.12	-	
mutual ventral cradling	-.68***	-.59***	-.24***	-.08	.09	-

Note. Correlation matrix of the variables considered for factor analysis. *p < .05, **p < .01, *** p < .001

Appendix 2

Table 1. Factor scores from first factor analysis

Factor	*Attachment*	
	Item	Factor Loadings
	Approaches by Infant	.938
	Leaves by Infant	.888
	Rejections by Mother	.560
	Mutual Ventral Cradling	-.798

Note. The first factor analysis resulted in one emerging factor, explaining 65.49% of the variance. Based on the factor loadings, this factor was identified as *Attachment*.

Table 2. Factor scores from second factor analysis

Factor	*Attachment*		*Maternal Restraints*	
	Item	Factor Loadings	Item	Factor Loadings
	Approaches by Infant	.976	Restraints by Mother	-.564
	Leaves by Infant	.836		
	Rejections by Mother	.391		
	Mutual Ventral Cradling	-.716		

Note. The second factor analysis resulted in two emerging factors, explaining 74.65% of the variance. Based on the factor loadings after from Varimax rotation, Factor 1 was identified as *Attachment* and Factor 2 was identified as *Maternal Restraints*.
